# O.3.2-10 My Active Ingredient: making movement more accessible for people living with chronic conditions

**DOI:** 10.1093/eurpub/ckad133.155

**Published:** 2023-09-11

**Authors:** Jane Thornton

**Affiliations:** Fowler Kennedy Sport Medicine Clinic, Canada; Western Centre for Public Health and Family Medicine, Schulich School of Medicine and Dentistry, Western University, London, ON, Canada; Department of Epidemiology and Biostatistics, Schulich School of Medicine and Dentistry, London, ON, Canada; School of Kinesiology, Western University, London, ON, Canada

## Abstract

**Purpose:**

Physical activity can treat over 30 chronic conditions, yet four out of five Canadians are not active enough to get these benefits. Finding evidence-based physical activity guidance for specific chronic health conditions is challenging, and patients are unsure about what and how much exercise is needed. Healthcare providers can feel under-equipped to counsel on physical activity. In 2021, we created the Western Research Hub for Physical Activity and Health (the Hub), a team of 30 junior and senior scientists across seven Faculties at Western University (London, Canada) and a Community Advisory Council (CAC) made up of local organization representatives and patient partners. This collective works to lead and advocate for improved access for physical activity and its implementation.

**Project or policy description:**

Through ongoing discussion and regular meetings with The Hub, we learned about the barriers and opportunities patients face when approaching physical activity, and in response collaboratively developed My Active Ingredient (myactiveingredient.org – launch date June 3/2023), a peer-to-peer healthcare hub for movement as medicine, co-designed and curated by people living with chronic conditions, healthcare providers, and researchers. From 2021-2023, we held CAC meetings and Community Workshops to review resources and provide feedback on design and functionality of the site. We gathered information through small group discussion, online surveys, and the Patient Education Materials Assessment Tool to incorporate in the development of the website. We collect and create the best physical activity resources to help make movement more accessible for people living with chronic conditions, and support healthcare providers advising their patients. Our team updates the website on an ongoing basis with new research, resources, and programs related to physical activity identified by website users, the CAC, and/or researchers. The website will undergo a quarterly, two-part evaluation that focuses on website analytics (e.g., page views, sessions, traffic sources etc.) and usability (e.g., test with target representative users).

**Conclusions:**

My Active Ingredient will increase the accessibility and quality of physical activity advice and resources for individuals living with chronic conditions or other barriers to physical activity, and become the “go-to” source for evidence-based guidance for patients, caregivers, and health providers.

